# Comprehensive Analysis of Potential Prognostic Values of ANGPTLs in Colorectal Cancer

**DOI:** 10.3390/genes13122215

**Published:** 2022-11-25

**Authors:** Yang Zhang, Xuyang Yang, Sicheng Liu, Zixuan Zhuang, Mingtian Wei, Xiangbing Deng, Ziqiang Wang

**Affiliations:** 1 Colorectal Cancer Center, Department of General Surgery, West China Hospital, Sichuan University, Chengdu 610041, China; 2 Research Laboratory of Cancer Epigenetics and Genomics, Frontiers Science Center for Disease-Related Molecular Network, Cancer Center, West China Hospital, Sichuan University, Chengdu 610041, China

**Keywords:** ANGPTLs, colorectal cancer, expression profiles, prognostic values, clinical application

## Abstract

Colorectal cancer (CRC) is one of the most common malignant tumors in the world. CRC recurrence and metastasis cause poor prognosis. ANGPTLs (angiopoietin-like proteins) are a family of proteins that are widely involved in metabolic disease and tumorigenesis. The roles of ANGPTLs in CRC are still controversial and deserve further research. In this study, several databases were employed to explore the expression profiles, prognostic values, genetic alterations, potential biological function, and immune infiltration correlation of ANGPTLs in CRC. The expression of *ANGPTL4* was significantly positively correlated with the stage of CRC. Therefore, cell and molecular experiments were further performed to explore the roles of ANGPTL4. Our results showed that the transcriptions of *ANGPTLs* in colon cancer and rectal cancer tissues were lower than those in normal tissues, but the protein expression varied among different ANGPTLs. In addition, the high expression of *ANGPTLs* led to a relatively poor oncological outcome. Specifically, the expression of *ANGPTL4* is significantly positively correlated with the stage of CRC. Further investigation revealed that *ANGPTLs* are mainly involved in signal transduction and the regulation of transcription, while KEGG pathway analyses demonstrated pathways in cancer. Additionally, we also observed that ANGPTL4 could promote the proliferation and migration of CRC cells, and four specific small molecule compounds had potential ANGPTL4-binding capabilities, suggesting the clinical application of these small molecule compounds on CRC treatment. Our findings imply the prognostic values and potential therapeutic targets of ANGPTLs in CRC.

## 1. Introduction

Colorectal cancer (CRC), ranking as the third most common malignant tumor in the world, is the second leading cause of cancer death [1]. In recent years, the prognosis of CRC in patients has been greatly improved because of the establishment of total mesorectal excision (TME) or complete mesocolic excision (CME) and the application of selective neoadjuvant chemoradiotherapy (nCRT) [2,3,4,5,6]. However, CRC recurrence and metastasis still cause a poor prognosis [7,8]. Tumor metastasis is a complicated process involving a series of epigenetic and genetic changes in the tumor cells and the tumor microenvironment [9,10]. Much is still unknown about the mechanism of CRC metastasis. Therefore, it is valuable to explore and clarify novel molecules associated with CRC metastasis.

ANGPTLs (angiopoietin-like proteins) are a family of proteins that are structurally similar to angiopoietins (ANGs) [11]. ANGPTL2 was the first to be reported in 1999 [12]. With a coiled-coil domain and fibrinogen-like domain that are conserved in ANGs, ANGPTL2 was initially named angiopoietin-related protein-2 (ARP2). They were proven to function and regulate angiogenesis in the vascular system [11]. Later, ANGPTLs were reported to be widely involved in metabolic disease and tumorigenesis [13,14,15,16,17]. Specifically, ANGPTLs participate in the regulation of cell adhesion, migration, and angiogenesis in CRC [15]. Generally, ANGPTLs promote CRC metastasis by inducing angiogenesis, such as ANGPTL2 [18] and ANGPTL6 [19]. However, ANGPTL1 could inhibit the migration, invasion, and stemness of colorectal cancer cells and further attenuate CRC liver metastasis [20,21]. In addition, ANGPTL7 was reported to play an antiangiogenic role and inhibit CRC liver metastasis [22]. Of note, ANGPTL4 was reported to be both a prometastatic [23,24,25,26] and antimetastatic [27] factor in CRC. Therefore, the roles of ANGPTLs in CRC are still controversial and deserve further research.

Eight ANGPLTs have so far been discovered, named ANGPTL1 to ANGPTL8 [28]. Seven ANGPTLs (ANGPTL1 to ANGPTL7) have been reported in CRC and collected in some databases [15]. Therefore, in this study, several public databases were used to explore the expression profile and prognostic values of ANGPTLs in human CRC. We also performed experiments to confirm the effects of ANGPTL4 on colon cancer cell lines. Our findings might reveal the potential application value of ANGPTLs in the treatment of CRC.

## 2. Methods and Materials

### 2.1. Gene Expression Profiling Interactive Analysis 2 (GEPIA2)

GEPAI2 (http://gepia2.cancer-pku.cn/#index accessed on 25 June 2022) is an online analytical tool for users to analyze RNA sequencing expression data. It contains data from thousands of tumors and normal tissue samples from The Cancer Genome Atlas (TCGA) and the Genotype-Tissue Expression (GTEx) websites [29]. In this study, GEPIA2 was used to analyze the mRNA levels of *ANGPTLs* between colon tumor/rectum tissues and normal colon/rectum tissues. Student’s *t*-tests were performed. |Log2FC| > 1 and *p* < 0.01 were considered significant. The mRNA levels of *ANGPTLs* among the colorectal cancer tissues with different clinical stages were also analyzed. Furthermore, a Kaplan–Meier curve was used to analyze overall survival (OS) and disease-free survival (DFS). The median was selected as the group cut-off for the survival plot.

### 2.2. The Human Protein Atlas

The Human Protein Atlas (https://www.proteinatlas.org/ accessed on 25 June 2022) is an online database to obtain access to human proteins that include immunohistochemistry (IHC) profiles and transcriptome profiles [30]. In this study, immunohistochemical images were downloaded to compare the expression levels of ANGPTL proteins between normal colon tissues and colorectal cancer tissues.

### 2.3. cBioPortal for Cancer Genomics

cBioPortal for cancer genomics (http://www.cbioportal.org/ accessed on 26 June 2022) is an online website that can be used to analyze gene alterations [31]. In our study, 4535 samples were selected to analyze *ANGPTLs* to reveal the proportion of genetic alterations and further divide them into 3 subtypes: mutation, amplification, and deep deletion.

### 2.4. String

String (https://cn.string-db.org/ accessed on 26 June 2022) is a database that provides protein–protein interactions (PPIs), which include physical and functional associations [32]. In this study, a PPI network was constructed to illustrate the interactions among ANGPTLs. In addition, another PPI network was constructed according to 100 co-expressed ANGPTL genes downloaded from GEPIA2.

### 2.5. Cytoscape

Cytoscape (https://cytoscape.org/ accessed on 27 June 2022) is a software project for visualizing and integrating networks [33]. As we mentioned above, two PPI networks were first constructed by STRING, and Cytoscape was used to beautify the networks. When constructing the network of the 100 genes co-expressed with ANGPTLs, the cytoNCA plugin was used. In detail, we ranked these genes by betweenness centrality (BC), which is an indicator showing the importance of nodes. A larger node size represents higher BC values in the interacting proteins. The top 1/4 proteins with BC values were placed in the inner circle.

### 2.6. DAVID

DAVID (Database for Annotation, Visualization, and Integrated Discovery) (https://david.ncifcrf.gov/ accessed on 28 June 2022) is an online website for functional annotation and gene lists [34]. In our study, DAVID was employed to analyze the Kyoto encyclopedia of genes and genomes (KEGG) pathway enrichment and gene ontology (GO) function of the 100 genes co-expressed with ANGPTLs.

### 2.7. TIMER2.0

TIMER2.0 (http://timer.cistrome.org/ accessed on 28 June 2022) is a public website to investigate the associations between immune infiltrates and genetic features [35]. In this study, TIMER2.0 was used to analyze the correlation between the expression level of ANGPTLs and four immune cell types in colon cancer and rectal cancer.

### 2.8. Protein Data Bank

The Protein Data Bank (PDB) (https://www.rcsb.org/ accessed on 1 July 2022) is an online database of the structural data of biological macromolecules [36]. In this study, the three-dimensional structure of the ANGPTL4 protein was downloaded from PDB, and four ligands were predicted to bind to the ANGPTL4 protein specifically [37].

### 2.9. Proteins Plus

Proteins Plus (https://proteins.plus/ accessed on 1 July 2022) is an online website focusing on the visualization of protein–ligand interactions [38]. As we mentioned above, the structure of the ANGPTL4 protein and its ligands were downloaded from PDB, and then Proteins Plus was used to predict their binding site. The DoGSiteScorer method was used to detect potential binding pockets of the ANGPTLs4 protein. DoGSiteScorer is a grid-based method to detect potential binding pockets based solely on the three-dimensional structure of the protein [39].

### 2.10. Cell Lines

Human colon cancer cells HCT116 and HT29 were purchased from the cell bank of the Chinese Academy of Science (Shanghai, China) and were cultured as previously described [40]. In brief, Dulbecco’s modified Eagle’s medium (DMEM) containing 10% fetal bovine serum (FBS) and 1% penicillin plus streptomycin was employed to culture these cells at 37 °C in a humidified atmosphere of 5% CO_2_. Tests were performed to confirm no mycoplasma contamination.

### 2.11. Plasmid Construction and Cell Transfection

The human *ANGPTL4* gene was amplified by PCR and cloned into the CMV-F vector. The recombinant plasmid CMV-*ANGPTL4* or empty plasmid CMV-F was transfected into human embryonic kidney cells (293T) and packaged out recombinant lentivirus by PEI. Then, HT29 or HCT116 cells were infected with lentivirus using 8 µg/mL polybrene. Next, HT29 or HCT116 cells were selected with puromycin (2.5 µg/mL) for 2 days. Finally, the overexpression efficiency was confirmed by Western blot.

### 2.12. Western Blot

The main procedure was performed as described in our previous report [41]. In brief, the total cell protein lysates were obtained by RIPA lysis buffer (50 mM Tris-HCl, 150 mM NaCl, 1 mM EDTA, 0.5% sodium deoxycholate, 0.1% SDS, 1% Triton X-100, pH 7.4) containing protease inhibitors. A total of 20 μg of protein lysates from each lane were separated by 10% sodium dodecyl sulfate–polyacrylamide gel electrophoresis (SDS–PAGE). Then, the proteins were transferred to PVDF membranes. After blocking with 5% nonfat dry milk, the membranes were incubated with primary antibodies. ANGPTL4 (A2011, 1:1000) and GAPDH antibodies (TA-08, 1:1000) were purchased from ABclonal and ZSGB-BIO, respectively. The anti-rabbit secondary antibody (ZB-2301, 1:5000) and anti-mouse secondary antibody (ZB-2305, 1:5000) were purchased from ZSGB-BIO.

### 2.13. Cell Proliferation

The Cell Counting Kit-8 (C0037, Beyotime, Hangzhou, China) was applied to assess the cell proliferation ability of HT29 and HCT116 according to the manufacturer’s protocol.

### 2.14. Transwell Migration Assay

Transwell migration assays were used to detect migration ability. In brief, 100 µL 2% FBS DMEM containing 1 × 10^5^ HT29 cells or 2 × 10^4^ HCT116 cells was seeded into the upper chamber of the transwell chambers (24 wells) (Corning, NY, USA). Then, 600 µL of DMEM containing 10% FBS were seeded into the lower chamber. After 48 h, the bottom surface of the membrane was fixed with paraformaldehyde, stained with crystal violet, and photographed. The number of migrating cells was counted in three random microscopic fields under an inverted microscope. Each assay was repeated at least three times. One representative of three independent experiments is shown.

### 2.15. Statistical Analyses

All statistical analyses were performed with GraphPad Prism 8.0 and R 4.1.3. Unpaired Student’s *t*-test was used to evaluate the difference between the two groups. *p* < 0.05 was statistically significant.

## 3. Results

### 3.1. Differential Expression of ANGPTLs in Patients with Colon Cancer and Rectal Cancer

**Figure 1:**
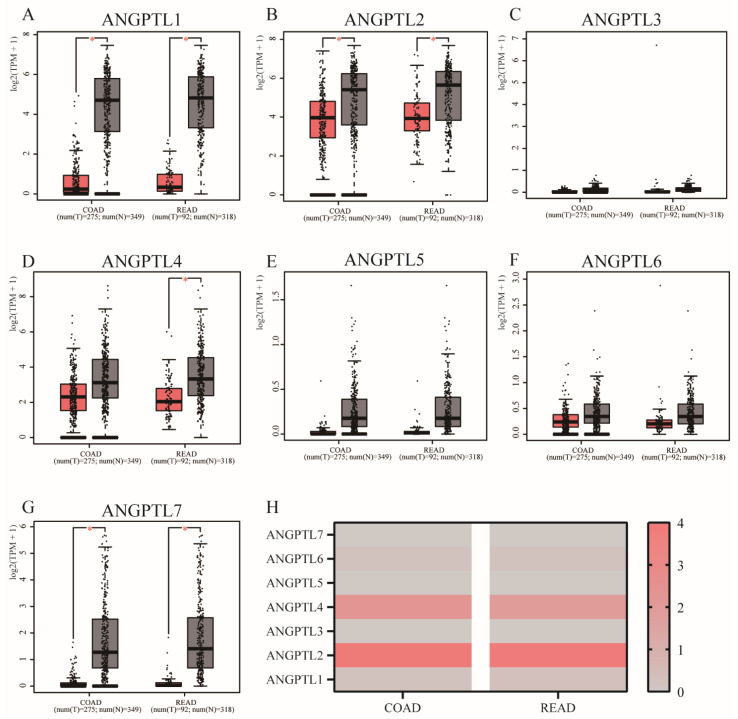
The mRNA expression levels of ANGPTLs in patients with colon cancer and rectal cancer (GEPIA2). (**A**–**G**) mRNA expression levels of seven ANGPTLs in colon/rectal cancer patients and normal tissues. T: colon/rectal cancer patient tissues; N: normal tissues. (**H**) The relative expression of seven ANGPTLs in colon/rectal cancer patients. * *p* < 0.05.

**Figure 2:**
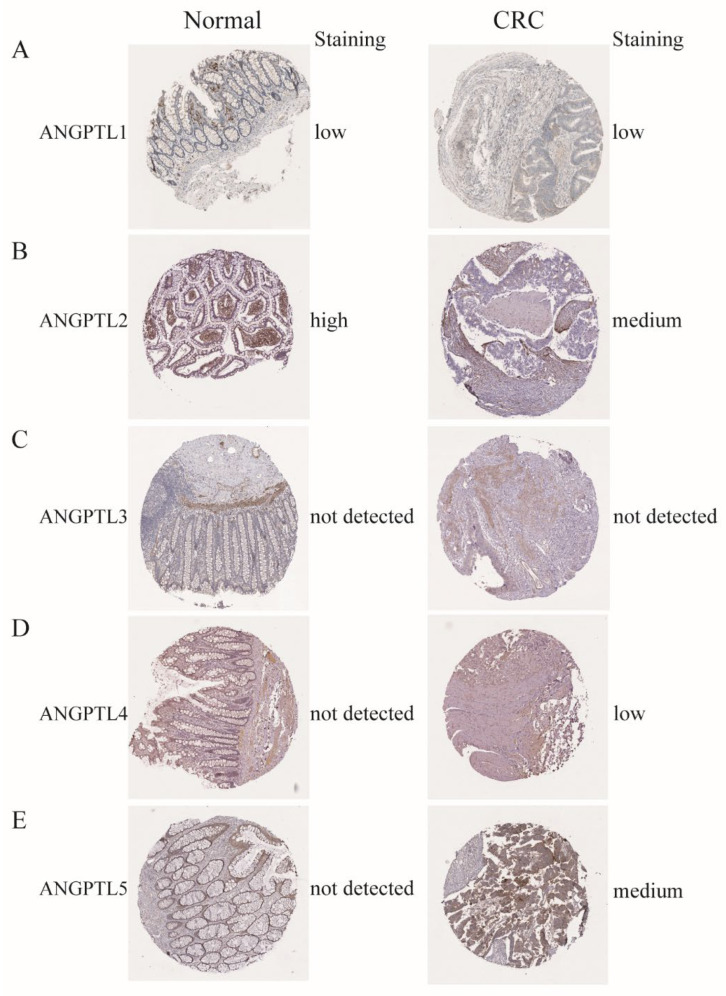
Representative immunohistochemistry images of ANGPTLs in patients with colorectal cancer (The Human Protein Atlas). (**A**–**E**) The protein expression profiles of five ANGPTLs in normal tissues and colorectal cancer tissues.

To investigate the differential mRNA expression of *ANGPTLs* in patients with colon cancer and rectal cancer, data were first acquired from GEPIA2. The mRNA expression levels of *ANGPTL1/2/6* were significantly reduced in both the colon and rectal cancer tissues (Figure 1A–G). The mRNA expression level of *ANGPTL4* was significantly downregulated in the rectal cancer tissues, but there was no significant difference in the colon cancer tissues. The relative expression of seven *ANGPTLs* in colon and rectal cancer patients is compared in Figure 1H, showing that the expression of *ANGPTL2* and *ANGPTL4* are relatively higher. Next, we further investigated the ANGPTLs protein expression in CRC patients and the IHC images acquired from The Human Protein Atlas. The protein expression levels of ANGPTL4/5 were higher in the CRC tissues, and ANGPTL2 was found to be lower in the CRC tissues (Figure 2). Overall, the transcriptions of ANGPTLs were lower in colon cancer and rectal cancer tissues than those in normal tissues. However, the protein expression levels vary among different ANGPTLs.

### 3.2. The Prognostic Value of ANGPTLs in CRC Patients

**Figure 3:**
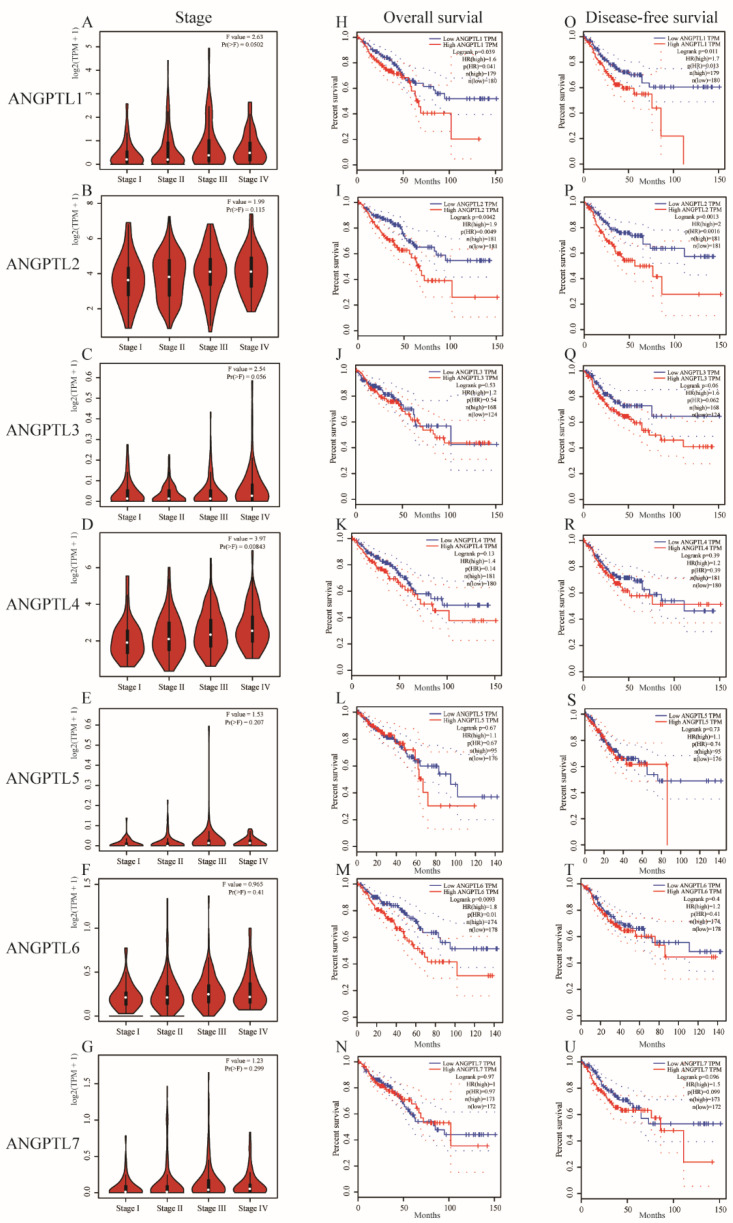
The prognostic value of ANGPTLs in CRC patients (GEPIA2). (**A**–**G**) Correlations between the expression of ANGPTLs and tumor stage in colorectal cancer patients. (**H**–**N**) The overall survival (OS) in colorectal cancer patients based on the expression of ANGPTLs. (**O**–**U**) The disease-free survival (DFS) in colorectal cancer patients based on the expression of ANGPTLs.

To investigate the prognostic value of ANGPTLs, we first studied the correlations between the expression of *ANGPTLs* and the tumor stage in colorectal cancer patients. The expression of all *ANGPTLs* showed an upward trend with the clinical stage (Figure 3A–G). Of note, the expression of *ANGPTL4* was significantly positively correlated with the stage of CRC (*p* < 0.01).

In addition, we further investigated the correlations between the expression of ANGPTLs and oncological outcomes. Higher levels of *ANGPTL1/2/6* expression led to significantly worse overall survival (OS) (Figure 3H–N). Upregulated *ANGPTL1/2* led to significantly worse disease-free survival (DFS) (Figure 3O–U). Overall, high expressions of *ANGPTLs* lead to a relatively poor oncological outcome.

### 3.3. Genetic Alterations of ANGPTLs in CRC Patients

**Figure 4:**
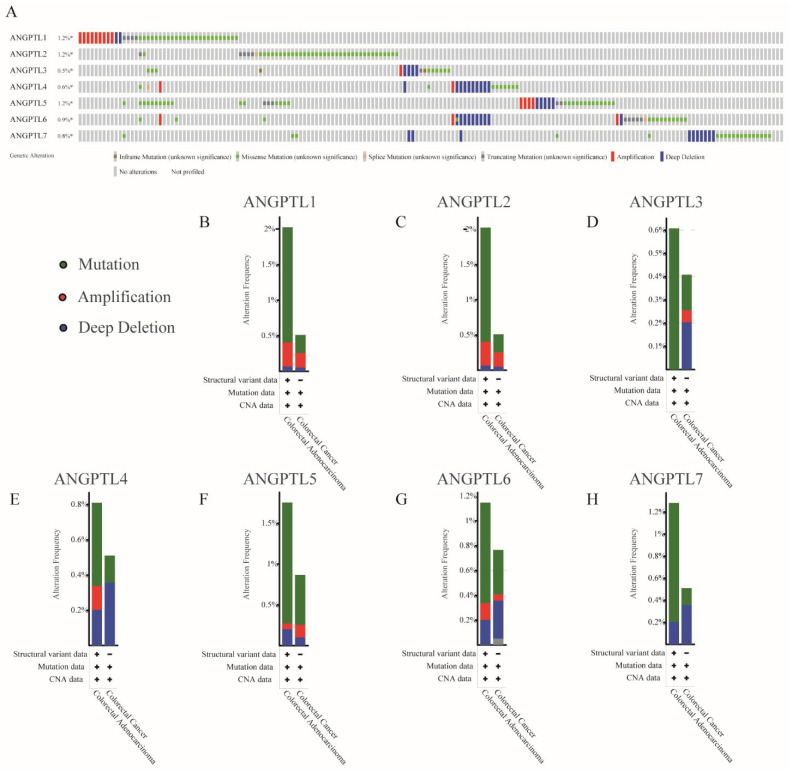
Genetic alterations of ANGPTLs in CRC (cBioPortal). (**A**) Summary of alterations of ANGPTLs in colorectal cancer, * represent the sample rate can represent the overall rate statistically. (**B**–**H**) Genetic alterations of seven ANGPTLs.

To study the genetic alterations of *ANGPTLs* in CRC patients, data were explored in cBioPortal for cancer genomics. The genetic alteration rates of *ANGPTLs* ranged from 0.5% (*ANGPTL3*) to 1.2% (*ANGPTL1/2/5*) (Figure 4A). Furthermore, genetic alterations of seven *ANGPTLs* were revealed (Figure 4B–F). Mutations accounted for the majority of genetic alterations for most *ANGPTLs*, which was more than the proportion for amplification and deep depletion. In conclusion, patients with genetic alterations of *ANGPTLs* only accounted for a small part of CRC patients, which might imply the universal applicability of *ANGPTLs* as therapeutic targets.

### 3.4. Functional and Interaction Analyses of ANGPTLs in CRC Patients

**Figure 5:**
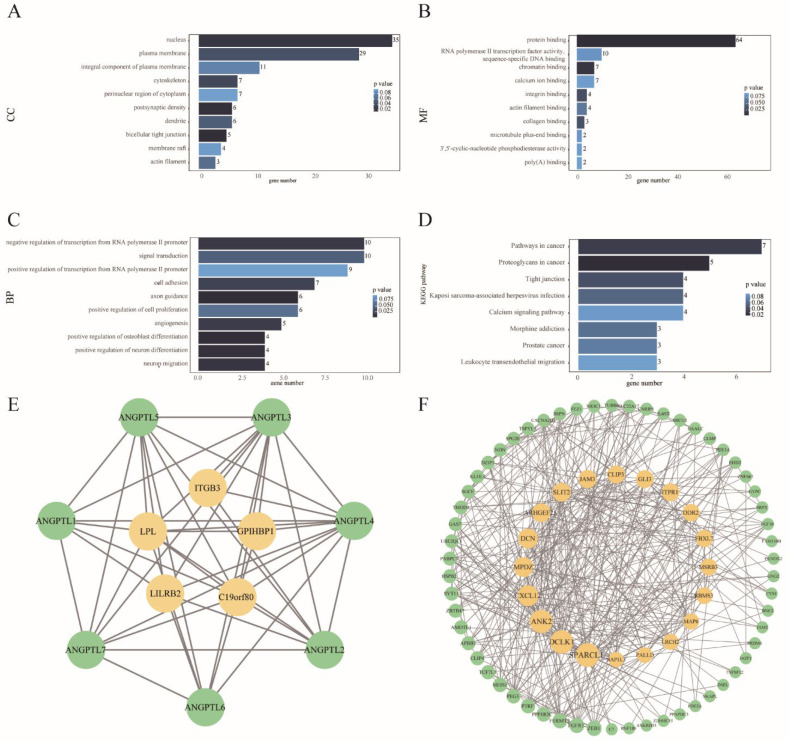
The function and interaction analyses of ANGPTLs in CRC patients (DAVID). (**A**–**D**) GO enrichment and KEGG pathway analysis of 100 co-expressed genes in ANGPTLs. (**A**) CC—cellular component. (**B**) MF—molecular function. (**C**). BP—biological process. (**D**) KEGG—Kyoto encyclopedia of genes and genomes. (**E**) Protein–protein interaction (PPI) enrichment analysis of seven ANGPTLs. (**F**) PPI enrichment analysis of 100 genes co-expressed with ANGPTLs.

To further study the function of *ANGPTLs*, 100 co-expressed genes were downloaded from GEPIA2. Next, DAVID was used to perform the GO function and KEGG pathway enrichment analyses. In cellular components (CC), these genes were mainly enriched in the nucleus and plasma membrane (Figure 5A–C). In the molecular function (MF), the majority of these genes function in protein binding. In the biological process (BP) category, these 100 co-expression genes were associated with the signal transduction and regulation of transcription. The KEGG pathway enrichment analysis showed that these co-expression genes were mainly involved in pathways in cancer (Figure 5D). In addition, two PPI networks were constructed by STRING and Cytoscape. LPL, ITGB3, GPIHBP1, C19orf80, and LILRB2 played vital roles in the interconnection among ANGPTLs. SPARCL1, DCLK1, ANK2, CXCL12, and MPDZ might be closely related to the function of ANGPTLs (Figure 5E,F).

### 3.5. Immune Cell Infiltration of ANGPTLs in CRC Patients

**Figure 6:**
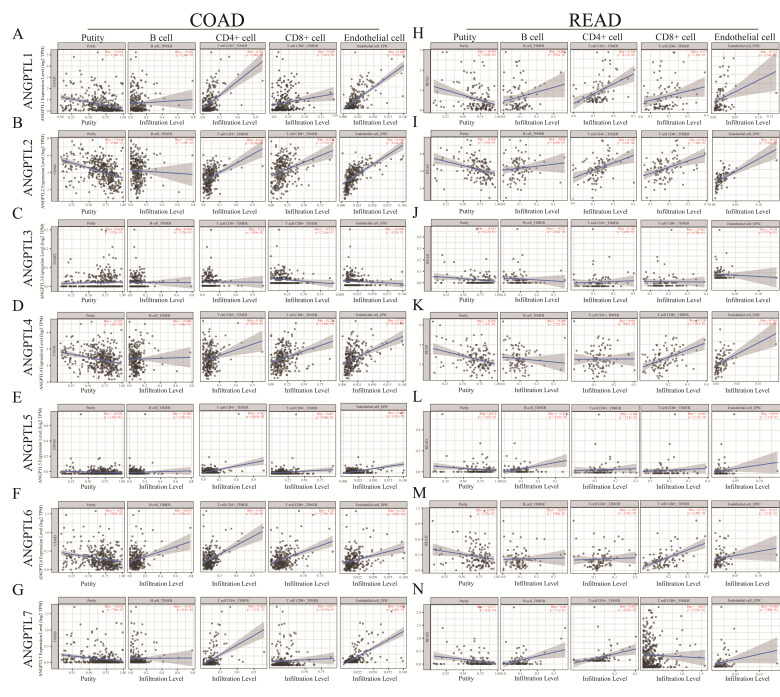
Correlations between seven ANGPTLs and immune cell infiltration in colon/rectal cancer (TIMER2.0). (**A**–**G**) Colon cancer. (**H**–**N**) Rectal cancer. Immune cells included B cells, CD4+ T cells, CD8+ T cells, and endothelial cells.

Immune cell infiltration in the tumor microenvironment (TME) has a great impact on tumor growth, angiogenesis, invasion, and metastasis [42]. Timer2.0 was used to analyze the correlations between the expression levels of different ANGPTLs and the immune cell infiltration in colon cancer and rectal cancer (Figure 6). Each dot in the scatter plots represents a single tumor sample. For example, ANGPTL4 is positively correlated with CD8+ T cells and endothelial cells in both colon cancer and rectal cancer. It is negatively correlated with B cells in rectal cancer and positively correlated with CD4+ T cells in colon cancer.

### 3.6. ANGPTL4 Promotes the Proliferation and Migration of CRC Cells

**Figure 7:**
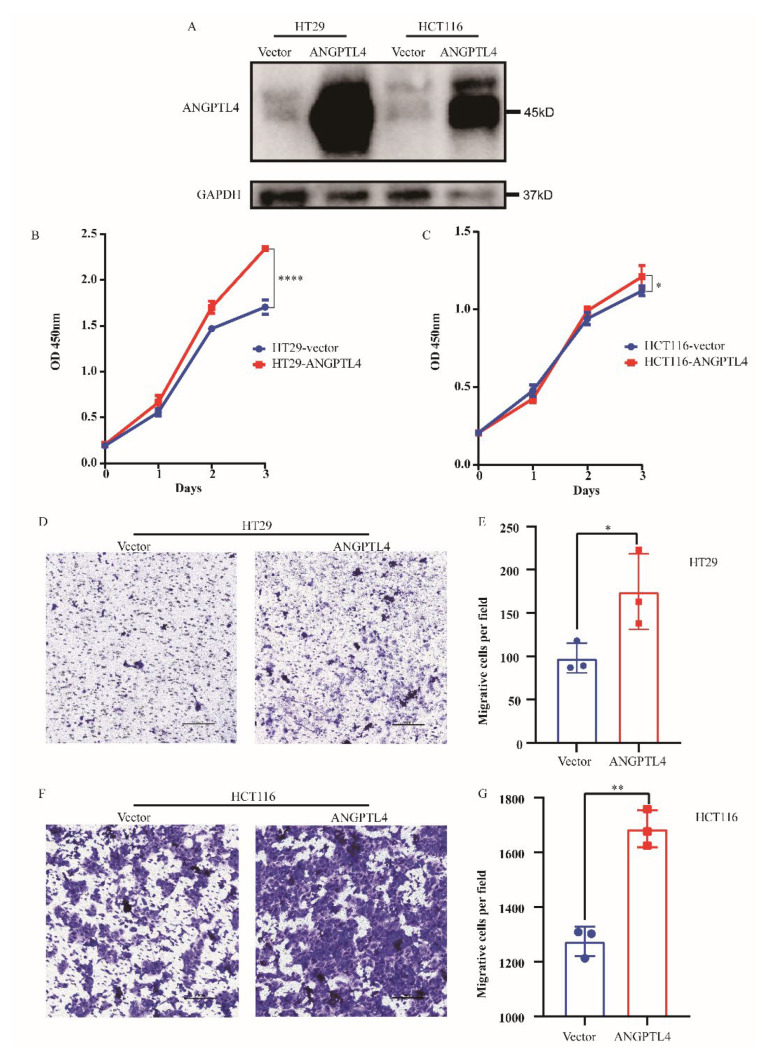
ANGPTLs4 promotes the proliferation and migration of CRC cells. (**A**) The protein level of ANGPTLs4 in HT29 cells and HCT116 cells after ANGPTLs4 overexpression. (**B**,**C**) The proliferation ability of HT29 and HCT116 cells measured by CCK8 assays. (**D**–**G**) The migration ability of HT29 and HCT116 cells measured by transwell migration assays. * represent *p* < 0.05; ** represent *p* < 0.01; **** represent *p* < 0.0001.

The above results revealed that *ANGPTL4* is significantly correlated with the stage of CRC, which suggests that ANGPTL4 might play a vital role in CRC progression and metastasis. However, this has not been proven by experiments. Therefore, we performed CCK8 assays and transwell assays to evaluate the proliferative and migrative ability of HT29 cells and HCT116 cells after transfection with *ANPTL4*. The overexpression efficiency of ANGPTL4 was first confirmed by Western blot (Figure 7A). Then, CCK8 assays and transwell assays revealed that overexpression of ANGPTL4 promoted the proliferation and migration of HT29 and HCT116 cells. Collectively, ANGPTL4 promotes the proliferation and migration of CRC cells (Figure 7B–G).

### 3.7. Potential Small Molecule Compounds Targeting ANGPTL4

**Figure 8:**
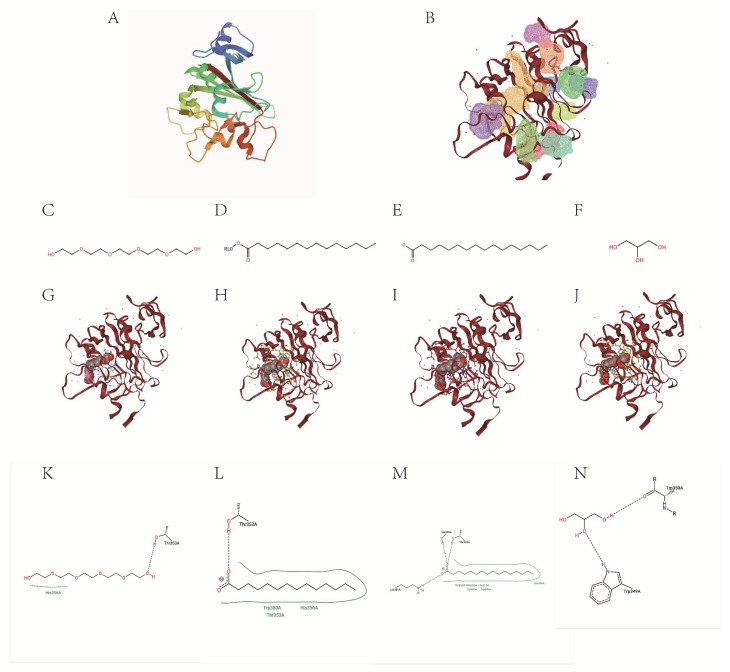
Potential small molecule compounds targeting ANGPTLs4. (**A**) The three-dimensional structure of ANGPTLs4 protein (Protein Data Bank). (**B**) Eleven predicted pockets of ANGPTLs4 (Proteins Plus). (**C**–**F**) The chemical formulas of four potential small molecule compounds targeting ANGPTLs4. (**G**–**J**) The three-dimensional structure revealing the combination of ANGPTLs4 and four potential small molecule compounds. (**K**–**N**) The two-dimensional chemical formulas revealing the combination. (**C**,**G**,**K**) Pentaethylene glycol. (**D**,**H**,**L**) Myristic Acid. (**F**,**I**,**M**) Palmitic Acid. (**F**,**J**,**N**) Glycerol.

We have proven that ANGPTL4 can promote CRC progression and metastasis and lead to poor prognosis in CRC patients. Therefore, we further investigated the potential small molecule compounds that could bind to ANGPTL4. The three-dimensional (3D) structure of the ANGPTL4 protein from the Protein Data Bank was revealed (Figure 8A). Then, we used the DoGSiteScorer method (Proteins Plus) to detect potential binding pockets in the ANGPTL4 protein. Eleven pockets of ANGPTL4 are also marked (Figure 8B). Furthermore, four specific ligands were predicted. Figure 8C–F shows the chemical structural formulas of these small molecule compounds (pentaethylene glycol, myristic acid, palmitic acid, and glycerol). The three-dimensional schematic diagrams of these ligands binding to ANGPTL4 (Figure 8G–J) and corresponding chemical structural formulas (Figure 8K–N) are also revealed.

## 4. Discussion

Cancer metastasis is the main cause of death in patients with CRC. The liver is the most common organ for CRC metastases. It was reported that approximately 50–60% of CRC patients would develop distant metastases, and 80–90% of these patients would lose the chances of surgery due to unresectable liver metastasis [43]. Improving the outcomes for these patients is still a tough challenge. For these patients with unresectable metastases, the National Comprehensive Cancer Network (NCCN) guidelines recommend chemotherapy plus targeted therapy. Bevacizumab, as a common anti-VEGF drug, is the first-line targeted drug for metastatic CRC patients [43,44]. However, the prognosis for such patients is still poor. The addition of bevacizumab could only increase the median overall survival time by approximately 1 to 5 months [45,46]. Although our previous study had performed an integrated analysis, which identified the potential hub genes serving as therapeutic targets and screened novel drugs in preventing CRC liver metastasis [47], the clinical practice is still limited. Therefore, it is necessary to further clarify the exact mechanisms of CRC metastasis and explore some novel molecules that might be applied for CRC targeted therapy.

ANGPTLs, short of angiopoietin-like proteins, are a family of proteins whose structures are similar to those of ANGs. ANGs mainly binds to Tie-2 tyrosine kinase receptors to exert crucial roles in angiogenesis [48]. However, previous studies considered ANGPTLs as ‘orphan ligands’ because no receptors were recognized. Later, Zheng et al. found immune-inhibitory receptors for several ANGPTLs, which could promote the expansion of blood stem cells and the progression of leukemia [49]. Recently, ANGPTLs were reported to participate in the process of cancer metastasis through the regulation of angiogenesis [11]. In this study, we reported that the transcriptions of ANGPTLs are lower in colon cancer and rectal cancer tissues than those in normal tissues, but the protein expression levels vary among different ANGPTLs. In addition, the high expression of *ANGPTLs* in CRC patients leads to a relatively poor oncological outcome. Specifically, the expression of *ANGPTL4* is significantly positively correlated with the stage of CRC. The GO enrichment analyses revealed that *ANGPTLs* mainly participate in the signal transduction and regulation of transcription, while KEGG pathway analyses demonstrated pathways in cancer. Furthermore, experiments were performed to prove that ANGPTL4 promoted the proliferation and migration of CRC cells. Four specific small molecule compounds were predicted to bind with ANGPTL4.

The survival analyses showed that high expression of *ANGPTLs* led to a relatively poor oncological outcome. In addition, the expression of *ANGPTLs* is positively correlated with the stage of CRC. Therefore, *ANGPTLs* could be recognized as oncogenes. Generally, oncogenes are more highly expressed in tumor tissues. However, our results revealed that the mRNA expression levels of most *ANGPTLs* were downregulated in the colon and rectal cancer tissues. We considered three possible reasons for this phenomenon. First, these genes might play different roles in the process of tumorigenesis and tumor progression. Second, transcriptome expression is usually measured by bulk sequencing, in which various cells are mixed. Therefore, the downregulated genes in the tumor tissues might not be lowly expressed in cancer cells. Third, the level of mRNA expression cannot always represent the protein level. There might be many posttranscriptional modifications. Some ANGPTLs are relatively highly expressed in the tumor tissue detected with IHC.

Given that the expression of *ANGPTL4* is significantly positively correlated with the stage of CRC, we assumed that ANGPTL4 might promote CRC metastasis. Therefore, CCK8 and transwell assays were performed to evaluate the proliferation and migration ability of the two CRC cell lines after being transfected with *ANPTL4*. The results showed that ANGPTL4 promotes proliferation and migration. Previous reports have shown that the roles of ANGPTLs are still controversial. ANGPTL4 was first reported to be an anti-angiogenic modulatory factor. Tube formation, corneal neovascularization, and miles permeability assays were used to prove that ANGPTL4 could suppress both angiogenesis and vascular leakiness [50]. However, another early study reported that ANGPTL4 could promote angiogenesis in ischemic tissues or solid tumor tissues (conventional renal cell carcinoma) due to the existence of hypoxia [51]. In addition, Chen et al. found that the knockdown of ANGPTL4 could inhibit proliferation and invasion in gastric cancer cells [52]. Meanwhile, the literature has reported on the roles of ANGPTL4 in CRC. Reports about the functions of ANGPTL4 in CRC are inconsistent. Kim et al. reported that ANGPTL4 stimulated by PGE2 could promote CRC cell proliferation [23]. Another study also showed that ANGPTL4 promotes CRC metastasis by upregulating the expression of NADPH oxidase 4 [24]. These results are in agreement with our findings and two other studies [25,26]. However, a recent study revealed that *ANGPTL4* knockdown stimulated colorectal cancer metastasis, suggesting that ANGPTL4 plays an antimetastatic role in CRC, which is in contrast to our results and the above literatures [27]. Intriguingly, a clinical research study revealed that ANGPTL4 was significantly lower in the plasma of colorectal cancer patients than in their normal counterparts [53]. However, this does not prove that ANGPTL4 is a tumor suppressor gene, as we mentioned above. Furthermore, ANGPTL4 was reported to be involved in metabolic diseases [54]. ANGPTL4 inhibits low-density lipoprotein (LDL) activity and increases circulating triacylglycerol (TAG) levels [55]. It was also proven to increase glutamine consumption and fatty acid oxidation in non-small cell lung cancer (NSCLC) [16].

To reveal the potential application value of ANGPTL4, we further investigated four potential small molecule compounds (pentaethylene glycol, myristic acid, palmitic acid, and glycerol) that could bind to ANGPTL4. Three-dimensional schematic diagrams of these ligands binding to ANGPTL4 were also shown. These small molecule compounds might be used as targeted drugs to treat CRC by binding to ANGPTL4. ANGPTL4 has indicated the potential to treat cardiovascular disease [56], hyperlipidemia [57], diabetic eye disease [58], and metabolic syndrome [59]. The application of ANGPTL4 as a cancer treatment target needs further research.

There were some limitations to this study. Our findings are mostly from online databases, and more in vivo animal experiments and in vitro cell and molecular experiments are required to further reinforce the findings and conclusions. Nevertheless, our findings might imply the prognostic values and potential therapeutic targets of ANGPTLs in CRC.

## 5. Conclusions

In conclusion, this study revealed that the transcriptions of ANGPTLs are lower in colon cancer and rectal cancer tissues than those in normal tissues, but the protein expression levels vary among different ANGPTLs. In addition, the high expression of *ANGPTLs* leads to a relatively poor oncological outcome. Specifically, the expression of *ANGPTL4* is significantly positively correlated with the stage of CRC. The GO enrichment analyses revealed that *ANGPTLs* are mainly involved in the signal transduction and regulation of transcription, while KEGG pathway analyses demonstrated pathways in cancer. Furthermore, experiments were performed to prove that ANGPTL4 could promote the proliferation and migration of CRC cells. Four specific small molecule compounds were predicted to bind with ANGPTL4. These small molecule compounds might be used as targeted drugs to treat CRC by binding to ANGPTL4. Our findings might imply the prognostic values and potential therapeutic targets of ANGPTLs in CRC. More in vivo and in vitro experiments are required to further validate these findings.

## Data Availability

All data generated or analyzed during this study are included in this published article. For further inquiries, please contact the corresponding author (wangziqiang@scu.edu.cn).
